# Clinical and Forensic Signs Resulting from Exposure to Heavy Metals and Other Chemical Elements of the Periodic Table

**DOI:** 10.3390/jcm12072591

**Published:** 2023-03-29

**Authors:** Carolina de Carvalho Machado, Ricardo Jorge Dinis-Oliveira

**Affiliations:** 1 Department of Public Health and Forensic Sciences, and Medical Education, Faculty of Medicine, University of Porto, 4200-319 Porto, Portugal; 2 TOXRUN—Toxicology Research Unit, University Institute of Health Sciences (IUCS), CESPU, 4585-116 Gandra, Portugal; 3 UCIBIO-REQUIMTE-Applied Molecular Biosciences Unit, Laboratory of Toxicology, Department of Biological Sciences, Faculty of Pharmacy, University of Porto, 4050-313 Porto, Portugal; 4 MTG Research and Development Lab, 4200-604 Porto, Portugal

**Keywords:** chemical elements, heavy metals, intoxication, pathophysiology, signs and symptoms, clinical and forensic diagnosis

## Abstract

Several heavy metals and other chemical elements are natural components of the Earth’s crust and their properties and toxicity have been recognized for thousands of years. Moreover, their use in industries presents a major source of environmental and occupational pollution. Therefore, this ubiquity in daily life may result in several potential exposures coming from natural sources (e.g., through food and water contamination), industrial processes, and commercial products, among others. The toxicity of most chemical elements of the periodic table accrues from their highly reactive nature, resulting in the formation of complexes with intracellular compounds that impair cellular pathways, leading to dysfunction, necrosis, and apoptosis. Nervous, gastrointestinal, hematopoietic, renal, and dermatological systems are the main targets. This manuscript aims to collect the clinical and forensic signs related to poisoning from heavy metals, such as thallium, lead, copper, mercury, iron, cadmium, and bismuth, as well as other chemical elements such as arsenic, selenium, and fluorine. Furthermore, their main sources of occupational and environmental exposure are highlighted in this review. The importance of rapid recognition is related to the fact that, through a high degree of suspicion, the clinician could rapidly initiate treatment even before the toxicological results are available, which can make a huge difference in these patients’ outcomes.

## 1. Introduction

Acute and chronic poisoning from exposure to various chemical elements can have significant morbidity and mortality [1]. Therefore, clinicians should be aware of those elements that can cause significant harm to patients if not detected and treated quickly. When intoxication is suspected, the diagnosis is based on a careful history of exposure, physical examination, and laboratory tests to render the best care [1,2,3]. Heavy metals such as lead, cadmium, mercury, and metalloid arsenic are among the chemical elements that may pose the greatest threat to human health. Heavy metals are usually defined as those having a density of more than 5 g/cm^3^; however, there is no consensual definition [4]. Although their reactivity is the reason why they may cause toxicity, it is also essential to vital physiological processes. Examples of essential elements include iron in oxygen transport, zinc in metabolism, and manganese and selenium in stress oxidative defense. Others, such as cadmium and bismuth, do not have a recognized useful biological role, being mostly considered toxic [3].

Although most intoxications occur due to environmental and/or occupational accidental exposures, chemical elements poisonings can also result of a suicide/homicide attempt. Metals such as lead and mercury, due to their concentrations in the environment, are more likely to result from environmental exposure [5,6,7,8]. On the other hand, arsenic and thallium are two chemical elements known for their use in criminal poisonings [1,9,10,11,12,13]. This reveals the importance of gathering a detailed history of the patient since it can be essential in the diagnosis of a condition that easily raises suspicion of other pathologies, particularly abdominal and neuropsychiatric ones.

Previous studies by our research group have highlighted the relevance of image in the clinical and forensic suspicion and presumptive diagnosis, and different aspects of intoxications have been reviewed in the last few years [14,15,16,17,18]. This work follows this major objective by fully reviewing the pathophysiology, clinical presentation, forensic features, and probable sources of exposure to different chemical elements, such as heavy metals, including thallium, lead, copper, mercury, iron, cadmium, and bismuth, as well as other chemical elements such as arsenic, selenium and fluorine. Some of these characteristic signs may lead to the suspicion of a specific element, and consequently to the early detection of the underlying intoxication as well as the early formulation of emergency treatment. As Paracelsus noted, “Poison is in everything, and nothing is without poison. The dosage makes it either a poison or a remedy” [19].

## 2. Materials and Methods

The electronic search was conducted using PubMed and Google Scholar concerning signs and symptoms, history and physical examination, pathophysiology, clinical and forensic diagnosis of intoxications by heavy metals such as thallium, lead, copper, mercury, iron, cadmium, and bismuth, as well as other chemical elements such as arsenic, selenium and fluorine. The keyword “intoxication” was crossed with thallium, arsenic, lead, fluorine, copper, selenium, mercury, iron, cadmium, and bismuth, as well as their respective forensic and clinical signs. Furthermore, retrieved journal articles, governmental documents, and books were reviewed to expand the sources of information. This research was extended to articles written in all languages. Scientific documents, including books, articles, and government documents were included in this review.

## 3. Thallium

Thallium (Ti) is a toxic metal accidentally discovered in 1861 due to the dust burning from a sulfuric acid industrial plant. This prompted the sighting of a bright green spectral band that quickly vanished [2,20]. In the past, thallium was mainly used as a rodenticide [21], but as the number of accidental intoxications increased, several countries banned this type of usage. The use of thallium was also discontinued in the treatment of syphilis, gonorrhea, tuberculosis, and trichophytosis [22]. Before 1930, scalp ringworm treatments made use of thallium’s depilatory properties until reports of pediatric deaths led to the clinical abandonment of the mentioned approach [23]. Modern thallium usage includes the manufacturing of green-colored fireworks, imitation jewelry, optical lenses, semiconductors, low-temperature switching devices, and scintillation counters by acting as a chemical catalyst and as a component of artistic paints. The metal’s lethal dose is approximately 10–15 mg/kg [22,23]. On the medicine realm, it is used in the perfusion scintigraphy of the myocardium and in the detection of certain malignant tumors [22]. Given its rapid myocardium distribution and uptake, small nontoxic dosages of radioactive thallium are still used today in cardiac dysfunction spotting [24].

Thallium salts lack taste and odor, while also having the ability to completely dissolve in liquids, being absorbed at a fast speed and evade detection on routine toxicological reports. Such properties make it a perfect candidate for criminal poisonings [9,10,11]. Nevertheless, such occurrences are rather rare in most Western societies [24]. The poisonings essentially occur from salt ingestion, but there have been reports of dust or fume inhalation from smelting, skin absorption, and even overdose from cocaine and heroin consumption [25,26]. Upon exposure, thallium is rapidly distributed to all body tissues. Association with the Na^+^/K^+^-ATPase channel occurs at 10 times the affinity rate of potassium, which results in its binding activity being interrupted. Once thallium’s intracellular stores amass, interference with the proper function of several enzymes occurs by binding sulfhydryl groups located on the mitochondrial membrane, which inhibits cellular respiration, disrupts calcium homeostasis, and interact with riboflavin and riboflavin-derived cofactors. Additionally, thallium binds to glutathione, which inhibits its activation as well as the ability to metabolize heavy metals, causing them to over accumulate in the body [27].

**Figure 1:**
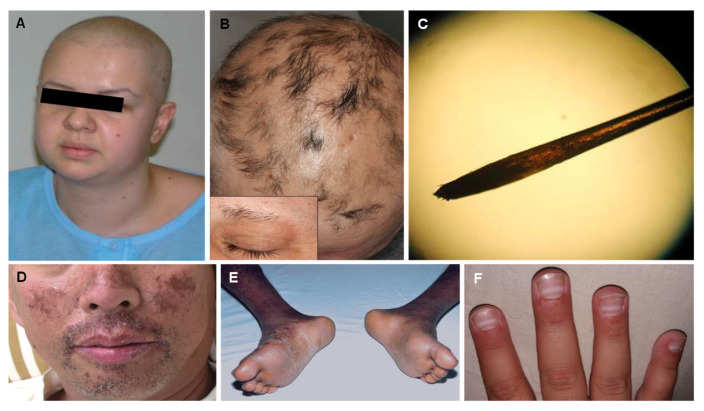
Characteristic scalp hair loss (**A**,**B**), blackened hair root under light microscopy (**C**), acne-form pustular lesions, lip oedema, and angular stomatitis (**D**), keratosis of soles (**E**), and Mee’s lines (**F**). Reprinted from (**A**)—[28], (**B**)—[38], (**C**)—[1], (**D**)—[36], (**E**)—[39], (**F**)—[40].

Prompt diagnosis of thallium poisoning can be complicated due to the variety of non-specific symptoms. Acute polyneuropathies (i.e., painful paresthesia of hands and legs, particularly the soles of the feet, and distal weakness predominantly in the legs), gastrointestinal symptomatology (i.e., vomiting, diarrhea, abdominal pain, or obstipation), and later alopecia and other dermatological signs must imply the consideration of thallium poisoning [28,29]. Both peripheral neuropathy and gastrointestinal signs are early features of thallium poisoning [30]. The initial dermatological symptoms are not specific to thallium poisoning. Such symptoms include skin desquamation with keratosis on palms and soles (Figure 1E), acne-form or pustular eruptions on the face, angular stomatitis (Figure 1D), and painful glossitis with tongue tip [31]. The typical triad unfolds within 2–3 weeks [23] with the appearance of dermatological signs such as the loss of scalp hair (Figure 1A,B) and the lateral aspects of eyebrows with relative preservation of the eyelashes, pubic hair, and axillary hairs [20] and, at the same time, transverse white lunar stripes on nails (Mee’s lines or Aldrich–Mee’s lines (Figure 1F)) [32]. Acute hair loss seems to occur due to the binding between thallium and the cysteine sulfhydryl groups found in hair, while dermatitis, Mees’ lines and neuropathy are likely effects of secondary riboflavin deficiency [20,24,33,34].

A significant feature of acute thallium poisoning is the presence of tapered or bayonet hair (Figure 1C), an abnormal anagen hair with a tapered dystrophic root [35,36] as well as darkened hair roots when examined under a light-powered microscope. In humans, this occurrence is reported to be detected as early as 4 days after poisoning [37] and illustrates an optical phenomenon which is caused by the accumulation of gaseous inclusions responsible for light diffraction. This finding can be highly suggestive to thallium poisoning diagnostic before the onset of alopecia [35].

## 4. Arsenic

Arsenic (As) is an abundant metalloid found throughout the Earth’s crust and soil, and contamination can occur mainly due to water runoff and leaching [3]. Early reports of its usage date back to 400 B.C. by Greek and Roman physicians [41] and is still used in traditional Chinese and Indian folk medicine [42,43]. Western societies have recently used it as treatment for late-stage African trypanosomiasis and acute promyelocytic leukemia [3]. It is used in mining operations (for smelting), ceramic manufacturing, as pesticide for agricultural purposes, and in semiconductors and lasers in the electronics industry [44,45,46].

Arsenic compounds occur in three oxidation states: elemental, trivalent arsenite and pentavalent arsenate. While elemental is nontoxic, arsenite has ten times more toxicity potential than arsenate. This metalloid occurs in three chemical forms: organic, inorganic, and arsine gas. Organic arsenic shows little acute toxicity, whereas inorganic arsenic and arsine gas are highly toxic [47]. There is a natural arsenic occurrence in seafood that has nontoxic organic compounds, such as arsenobetaine, which can lead to higher urine arsenic levels [48,49]. Even considering that most acute arsenic poisoning occurrences are due to accidental ingestion of arsenic-containing pesticides and less commonly from attempted suicide or homicide [12], arsenic is still sometimes seen as the most common homicidal agent. Its historical use as a poison has earned it the title of “Poison of Kings and the King of Poisons” [13].

Although absorption by pulmonary and skin routes are possible, chronic arsenic exposure mostly occurs by the ingestion of geologically contaminated water. When chronic arsenic ingestion takes place, accumulation occurs predominantly in the liver, kidneys, heart, and lungs [50]. Even when most of the arsenic is eliminated from those organs, residual traces remain in the keratin-rich tissues such as nails, hair, and skin.

Arsenic reaction with the sulfhydryl groups in certain tissue proteins interferes with various enzyme systems vital to cellular metabolism [51]. The lethal dose of inorganic arsenic is approximately 0.6 mg/kg [12]. When acute arsenic toxicity occurs from ingestion, classic gastrointestinal symptoms emerge, including abdominal pain, nausea, emesis, and profuse watery or bloody diarrhea [13,46,52]. The mentioned symptoms are followed by hypotension, heart failure, pulmonary edema, and shock and can be seen as a consequence of cardiomyopathy, ventricular arrhythmias, and capillary dilation with fluid loss to third space [13,46]. Normally, peripheral neuropathy occurs 2 to 8 weeks after exposure, but it can also take place within hours of a severe exposure [13,53]. The latter consists a bilateral and symmetric sensorimotor neuropathy that can easily be misdiagnosed as the Guillain–Barré syndrome [13,54]. There are several malignancies associated with chronic arsenic exposure, namely in the skin, lung, liver, bladder, and kidney [55], with a higher prevalence in populations with occupational or environmental exposure to arsenic.

**Figure 2:**
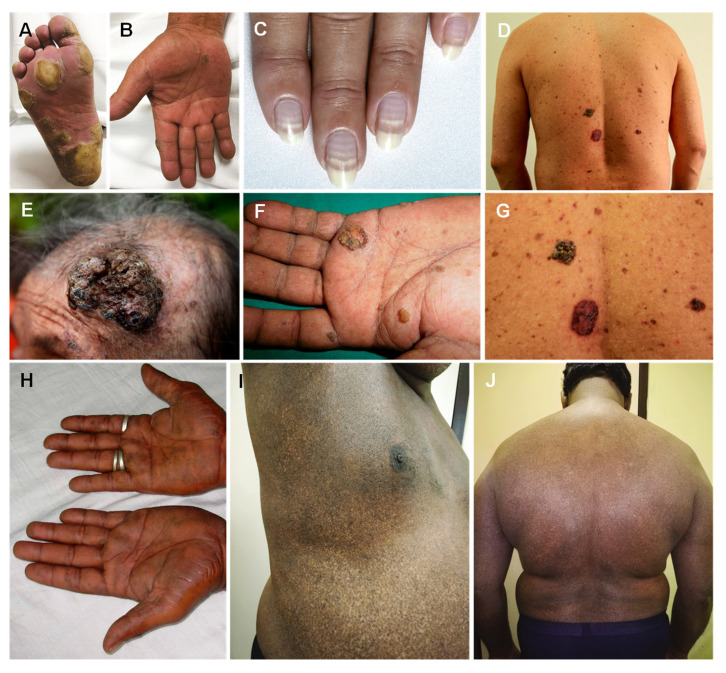
Hyperkeratosis of soles (**A**) and palms (**B**), Mee’s lines (**C**), diffused hyperpigmentation on the trunk (**D**), Bowen’s disease on head (**E**) and palm (**F**), hyperkeratosis on the trunk (**G**), palmar pits (**H**), and raindrop pigmentation (**I**,**J**). Reprinted from (**A**,**B**)—[70], (**C**)—[71], (**D**,**E**,**G**)—[72], (**F**,**H**)—[69], (**I**,**J**)—[73].

High levels of arsenic, either through ingestion or inhalation, results in acute symptoms of arsenicosis [56], which is defined by the World Health Organization working group [57] as a “chronic health condition arising from continuous ingestion (not less than 6 months) of arsenic above a safe dosage, usually manifested by characteristic skin lesions, with or without the involvement of internal organs”. Within 1 to 4 weeks, several cutaneous manifestations appear, having been described as ranging from raindrop pigmentation to fine freckles of spotted pigmentary changes (Figure 2H–J) [58,59,60] or hypopigmented lesions on the trunk and extremities, and mild to severe diffused hyperpigmentation or melanosis (Figure 2D,G) [61,62]. While most intense on the trunk, pigmentation can delocalize or diffuse, with a particular effect on skin folds. Macular areas of depigmentation may occasionally appear on normal skin or hyperpigmented background, known as leucomelanosis. Mucous membranes, such as the undersurface of the tongue or buccal mucosa, may develop a characteristic pigmentation: a blotchy pigmentation [58,59,60]. Roughly 5 weeks after arsenic exposure, the appearance of a transverse white stria, 1–2 mm in width, might occur above the lunula of each fingernail (Mee’s lines (Figure 2C)). This feature is a consequence of the nail matrix interruption, can be seen in acute and chronic poisoning, and is not arsenic pathognomonic [63]. The earliest and most common of several cutaneous features is melanosis [64,65]. Hyperkeratosis of palms (Figure 2B) and soles (Figure 2A) is considered to be pathognomonic of chronic arsenicosis, typically appearing after a prolonged ingestion of arsenic [66]. In a study by Rattner et al. [67], for an exposure of 4.75 mg/day, hyperpigmentation appeared after six months and hyperkeratosis occurred 3 years after.

Depending on the extent and severity, keratoses may be classified as mild, moderate, or severe [68]. When it reaches the severe form, it may form keratotic elevations more than 5 mm in size, which are considered as an early marker of carcinogenicity. Exposure to arsenic is related to three types of skin cancer, namely, Bowen’s disease (Figure 2E,F), basal cell carcinoma, and squamous cell carcinoma [69].

## 5. Lead

Lead (Pb) is a heavy metal known in Latin as *plumbum* (i.e., liquid silver), which historically led to the terms plumbism and saturnism for intoxications [74,75,76]. It is speculated that several Roman leaders were victims of lead poisoning and consequently suffered from neurotoxicity and sterility. The beginning of the industrial revolution led to an increase in the consumption of leaded products, mainly gasoline and lead-based paints [77]. In England, an outbreak of lead toxicity arose in 1700, caused by lead contaminated cider. The victims developed intense abdominal pain and were said to have “Devonshire Colic” [78]. Benjamin Franklin described in 1973 the abdominal pain and peripheral neuropathy caused by lead [79]. Although it was not yet extinguished, lead poisoning began to decline progressively in developed countries due to an increased surveillance of industrial and domestic exposure to lead [80]. In developing countries, by contrast, lead poisoning is still present, and, on a worldwide scale, continues the most frequent of occupational poisonings [6]. Exposure is primarily via the respiratory tract [7,8]; however, in pediatric plumbism, the “pica syndrome” is usually the common denominator [81,82,83].

The manufacture of lead batteries, colored paints, lead compounds, and rubber and glass products can create enough lead dust in the air to cause poisoning. In the demolition industry, poisoning by inhalation of lead oxide fumes can occur. Gun club members and instructors show an increased lead absorption due to exposure to lead dust and lead oxide fumes from bullets and explosive charges, respectively. The application of lead-based paints as well as the manufacture of polyvinyl chloride (PVC) plastics is also a form of lead exposure, although final materials are completely safe [84].

Lead is an electropositive metal that presents high attraction for sulfhydryl groups and thus inhibits sulfhydryl-dependent enzymes, particularly intracellular calcium channels. This results in a deficiency in heme production, proximal renal tubular, and osteoblast dysfunction. Among others, lead also affects the vasomotor action of smooth muscle as a result of its effect on Ca^2+^-ATPase, which can produce abdominal pain [85]. Lead can deteriorate the integrity of the blood–brain barrier by breaking the intracellular junction of capillary endothelium. This increases the capillary leak into the central nervous system and, consequently, an increase in the intracranial fluid. This metal can also affect several neurotransmitters, namely acetylcholine, γ-aminobutyric acid (GABA), and dopamine, causing their spontaneous release. This causes N-methyl D-aspartate (NMDA) glutamate receptors to be blocked and an increase in protein kinase C levels [86,87].

**Figure 3:**
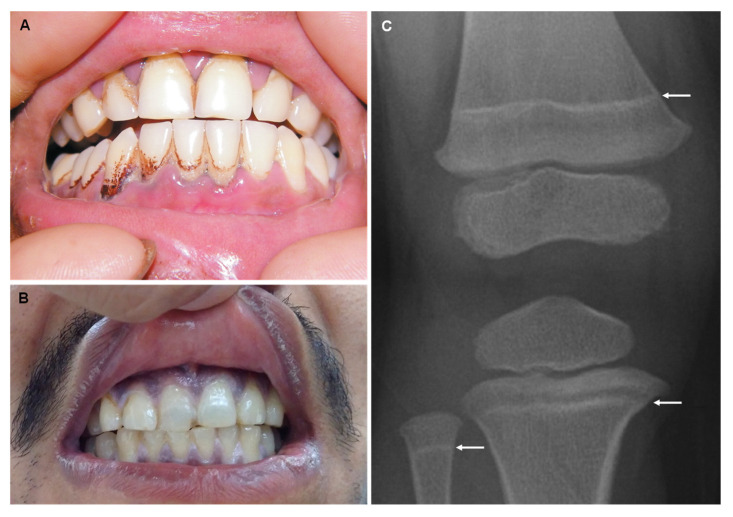
Characteristic Burton’s lines are highlighted by the deposits (**A**) and blue-purplish discoloration of the gums (**B**). X-ray with arrows showing “lead lines” (**C**). Reprinted from (**A**)—[94], (**B**)—[95], (**C**)—[96].

Any trail of lead in the human body can be seen as contamination since there is no known physiological role. The United States Centers for Disease Control and Prevention established in 2012 the average level of lead in the blood for adults to be 10 μg/dL and for children to be 5 μg/dL [88]. The main symptoms in young children (i.e., population particularly exposed) are irritability, loss of appetite, weight loss, behavioral and learning difficulties, abdominal pain, vomiting, constipation, anemia and renal failure [89]. The standard features to identify lead intoxication involve abdominal pain, anemia with basophilic stippling of red cells, blue-black gum deposits (Figure 3A,B), and a lead line on joint radiography (Figure 3C) [90]. The blue-purplish lines on the gums are known as “Burton’s lines”. These are created by a reaction between circulating lead and sulfur ions released during oral bacterial activity, which leaves lead sulfide at the junction of teeth and gums, creating deposits [91].

In some cases, radiographic imaging helps assist the diagnosis of lead poisoning and helps to clarify the etiology of exposure. In a patient with a potential ingestion of a lead-containing object, an abdominal X-ray is an important exam to be performed as well as in a patient with alleged plumbism or a history of bullet wound, on the region of bullet impact. Children between the ages of two and nine years old, when taking long bone radiographs, may exhibit increased metaphyseal density, also known as “lead lines”. Lead lines are transverse bands of increased density representing bone growth abnormalities because of lead exposure. This occurs due to the inhibition of the remodeling of calcified cartilage in the area of provisional calcification under the growth plate—chondral sclerosis. Multiple lines are a sign of repeated exposure to lead [92,93].

## 6. Fluorine

Fluorine (F) is the most electronegative element and the thirteenth most abundant occurring in rock phosphates, minerals, and the Earth’s crust in its ionic form. It is essential for the normal growth and development of various organs in our body, namely bone and teeth. For this reason, it is considered to be one of the essential microelements for the organism [97]. Since 1945, fluoride has been implemented as a supplement in several public drinking water systems in order to control dental decay [98]. In the last few decades, due to its success, the use of fluoride in different forms and concentrations for the prevention of dental caries has been increasing steadily [99]. Water fluoridation was named, by the US Centers for Disease Control, one of the 10 greatest public health achievements of the past century [100]. Fluorine impairs the enamel formation through the reduction in the calcium concentration in the matrix that ultimately affects protease activity, slowing down or blocking protein degradation in the enamel matrix. This impairment is dependent of the time and dose of exposure [101,102]. This can result in an interruption of enamel development and a consequent hypomineralization (increased porosity) of the enamel, allowing the appearance of white or yellowish lesions on the surfaces of the teeth [103]. In the skeleton, fluorine has a half-life of around 7 years. About 50% of the fluoride becomes incorporated in the hydroxyapatite crystals by replacing the hydroxide ions, thus altering the structure and size of the crystals. Furthermore, fluoride seems to influence bone turnover through its influence in the gene expression of RUNX family transcription factor 2 (Runx2) and the receptor activator of nuclear factor kappa-Β ligand (RANKL) [104]. In addition, it affects the expression of osteocalcin and osteoprotegerin and increases osteoblast activity [105,106].

**Figure 4:**
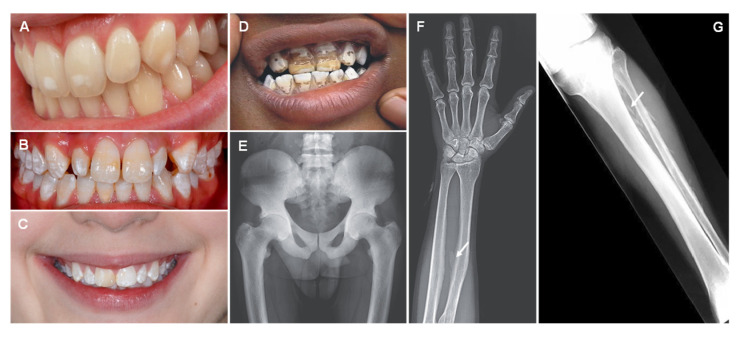
Dental fluorosis: mild (**A**), moderate (**B**,**C**), and severe (**D**). Pelvic radiography showing osteosclerosis (**E**). X-ray with arrows showing skeletal fluorosis evidenced by the diffuse bone condensation and interosseous membrane calcification of the forearm (**F**) and leg (**G**). Reprinted from (**B**)—[127], (**D**)—[128], (**E**,**F**)—[129], (**G**)—[126].

As fluoride has become substantially more included in dental products as well as in food sources (via fluoridated water), several sources of fluoride exposure are now related to the increased incidence of dental and skeletal fluorosis. When the fluoride level is higher than 1.5 mg/L (1.5 ppm) in drinking water, dental and skeletal fluorosis can occur [107]. Nutrition is equally crucial to controlling serum fluoride level, as ions such as calcium, magnesium, and aluminum can decrease fluoride bioavailability [108,109,110]. Dental fluorosis is reported to be the greatest risk of community water fluoridation [111,112]. Dental fluorosis, first described by Trendley Dean in 1937, is a developmental condition of enamel as a result of excessive absorption and repeated exposure to small doses of fluoride during all stages of tooth development [113,114]. The gravity of fluorosis observed is multifactorial, but is deeply connected with both the amount and timing of fluoride exposure [115,116]. Mild cases of dental fluorosis are clinically classified by a white opaque appearance of the enamel, as a result of increased subsurface porosity (Figure 4A). The first change is the appearance of thin white horizontal lines across the covering of the teeth, with white opacities at the recently erupted incisal end. The white lines appear along the ‘perikymata’, a term relative to horizontal ridges on the surface of the tooth, which correspond to the incremental lines in the enamel denominated as Striae of Retzius [117,118]. When exposed to higher levels of fluoride, white enamel lines become increasingly defined and thicker (Figure 4B,C). Some irregular cloudy sections and thick opaque bands also occur on the involved teeth. The aggravation of dental fluorosis can cause the entire tooth to turn chalky white and lose transparency. Deeper layers of enamel are affected when exposed to very long or very high doses of fluoride; the enamel becomes less well-mineralized [118,119]. In serious cases, porosity, pitting, and brown areas related to fragile enamel can frequently be noticed on the surface of the tooth (Figure 4D). Even so, an assertive diagnosis of the disease should only be reached from an adequate anamnesis and should not be confused with other types of discoloration and dental stains that present a very similar clinical picture, such as the hypomaturation type of amelogenesis imperfecta [120]. The upper incisors are especially vulnerable to the effects of fluorine, probably due to air exposure as a consequence of insufficient lip closure. As a result, the incisal section will become dried out for long periods, and any porosities will consequently be discerned. Additionally, the incisal edges/cusp tips do not overlay dentin, so any alteration in pore volume in these sections will reveal itself as clinically different from that of the remaining parts of the teeth. This can lead to the idea that the incisal section is more affected than the remainder of the surface, when in fact they are equally porous [121].

Severe juvenile skeletal fluorosis has also been reported by some authors who have associated it with an inadequate calcium intake in the diet [122]. Similar to dental fluorosis, skeleton fluorosis is caused by high levels of fluoride via ingestion or inhalation over a chronic period. High fluoride accumulation in bones and joints creates an imbalance in the bone mineral metabolism, resulting in bone reabsorption and abnormal levels of calcium (Figure 4E–G) [123,124,125]. Skeletal fluorosis is often asymptomatic. Nevertheless, as bones and joints become weaker, patients report pain in hands, feet and lower back, muscle weakness, chronic fatigue, and joint stiffness with the movement being difficult and painful [124,125]. Fusion of vertebrae, kyphosis with limited spinal mobility, flexion contracture of lower extremities, and restricted chest wall expansion may also be observed [124]. In severe cases, a progressive weakness of lower limbs, walking difficulty, and sphincter disturbances reveal the onset of radiculomyelopathy [126].

## 7. Copper

Copper (Cu) is a heavy metal that occurs in nature in the form of different minerals, chalcopyrite being the most abundant and economically significant. Copper deposits are classified according to how these deposits were formed. Porphyry deposits (found in mountainous regions of Western North and South America) are associated with igneous intrusions and represent two-thirds of the world’s existing copper [130,131]. The type of deposits found in sedimentary rocks (found in areas such as the Central African Copperbelt and the Zechstein basin of Eastern Europe) represent about a quarter of copper reserves [132]. Copper’s qualities, such as being easily stretched and shaped, resistant to corrosion, as well as able to conduct heat and electricity, make it a material of choice in a huge variety of domestic, industrial, and high-technology applications. Currently, copper is used in civil construction, power generation and transmission, manufacturing of electronic products, production of industrial machinery and transport vehicles, heating and cooling systems, telecommunications, motors, wiring, radiators, and touched surfaces (such as brass doorknobs), among others [133]. Furthermore, copper sulfate is commonly used in farming as a pesticide, in the leather industry, and in home-made glue, and burning copper sulfate is a common practice among Buddhists and Hindus [134]. Copper is also an indispensable element in the human body, acting as a cofactor for many enzyme pathways. This explains why copper homeostasis is extremely delicate. Copper toxicity is mainly related to its capacity to form reactive oxygen species [135,136,137].

Copperiedus (copper toxicity) can be primary, by a metabolic congenital defect, or secondary, as a result of high intake, increased absorption, or reduced excretion due to pathological processes [138]. In the secondary causes, poisoning may result from consuming acidic foods cooked in uncoated copper cookware, an exposure to excess copper in drinking water, copper salt-containing topical creams for burn treatments, through environmental sources, or in suicide attempts (the lethal dose is 10 to 20 g). Children are also often attracted to the bright color of the hydrated form of copper sulfate crystals, which leads to their intoxication [134]. Acute copper intoxication can present different symptoms such as metallic taste, nausea, vomiting, abdominal pain, heart failure, hepatic failure, renal failure, intravascular hemolysis, and ultimately death [139,140]. Wilson’s Disease is a congenital autosomal disorder of copper metabolism produced by a mutation in the ATP7B copper transporter gene and has a ratio of around 1/30,000–1/100,000 [141,142,143,144]. Mutations that result in a full absence or nonfunctional of ATP7B protein activity are rare and result in severe Wilson’s disease [145,146]. Chronically, excess copper will lead to a progressive deposition in the liver and brain, causing cirrhosis, acute liver failure, and nonspecific neuropsychiatric symptoms, such as dysarthria, dysphagia, tremors, ataxia, and the inability to concentrate [147].

**Figure 5:**
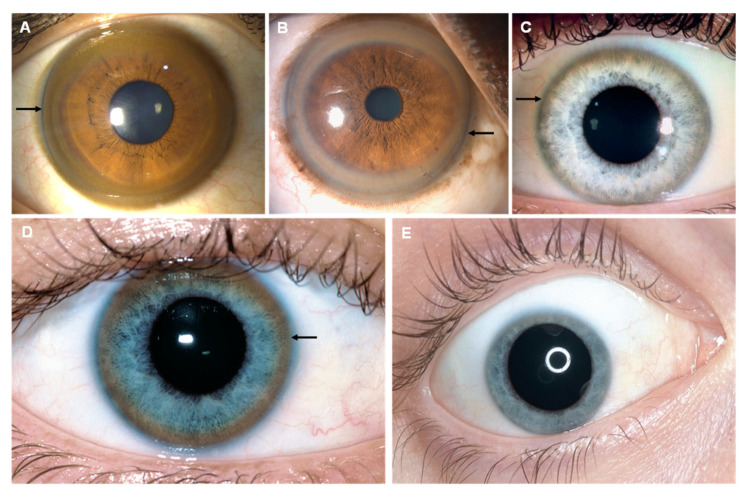
Kayser–Fleischer rings—(**A**–**E**). Reprinted from (**A**)—[164], (**B**)—[165], (**C**)—[166], (**D**,**E**)—[167].

**Figure 6:**
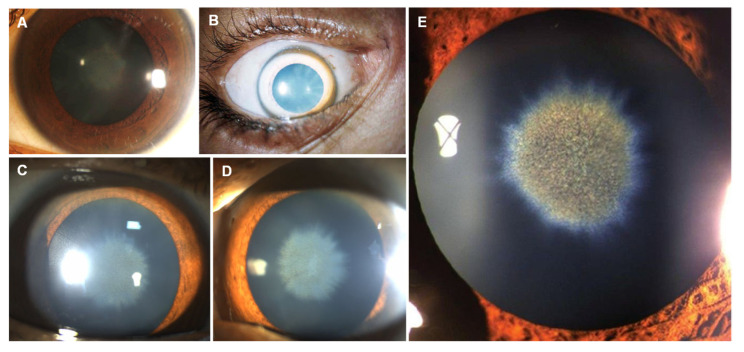
Sunflower cataracts seen with slit lamp—(**A**–**E**). Reprinted from (**A**)—[168], (**B**)—[169], (**C**,**D**)—[170], (**E**)—[171].

Copper deposition in the cornea creates the classical Kayser–Fleischer ring (Figure 5A–E). These rings are one of the few diagnostic indicators in clinical medicine. Kayser described the rings for the first time in 1902 and later Fleischer performed further studies in 1909 [148,149]. In 1949, Gerlach and Rohsrschneider finally established the fact that the rings were made of copper [150]. Kayser–Fleischer ring has a golden brown, green, or yellow coloration visible on the periphery of the cornea, caused by copper deposition in the Descemet’s membrane. In general, a slit lamp examination is essential for the confirmation. However, in some situations, it is visible to the naked eye. The Kayser–Fleischer ring occurs in more than 99% of patients with neuropsychiatric impairment, but barely in 25–50% of patients with liver disease or a pre-symptomatic state. The ring usually vanishes with treatment and its continuity or return implies a non-controlled copper state [151,152,153]. In 1922, Siemerling and Oloff [154] described an uncommon ocular manifestation of Wilson’s disease known as “sunflower cataract” (Figure 6A–E). They identified similarities between the lens changes seen in a patient with Wilson’s disease and those caused by an intraocular copper-containing foreign body [154]. Sunflower cataract incidence is lower than that of Kayser–Fleischer ring since sunflower cataracts are seen in the later stages of the disease and an attempted diagnosis of Wilson’s Disease decreases its incidence [155]. Sunflower cataract consists a thin, centralized opacification that is placed directly below the anterior capsule and encompasses between one-third and one-half of the anterior lens pole surface area. In all situations, the opacification is central and surrounded by other opacifications disposed in ray-like structures around it. This pattern, with a central disk encircled by petals, looks similar to a sunflower, justifying its designation [156,157,158]. Of note, sunflower cataract is not a “true” cataract, as it results from reversible copper deposition below the anterior lens capsule [154,159,160,161,162].

Although physicians can be guided toward a Wilson’s Disease diagnosis, in the presence of Kayser–Fleischer rings and sunflower cataracts, it cannot be considered a pathognomonic ocular sign of Wilson’s Disease due to its occurrence in other medical conditions, such as the presence of intraocular foreign bodies containing copper [156]. In opposition to the later, sunflower cataract in Wilson’s Disease is fainter and occurs in both eyes [163].

## 8. Selenium

Selenium (Se) is a non-metal chemical element and is fundamental to several biological human biological systems. Particularly, selenium is a fundamental trace element that constitutes more than two dozen selenoproteins that have a vital part in reproduction, thyroid hormone metabolism, deoxyribonucleic acid synthesis, and assurance from oxidative damage and infection [172]. The majority of water-soluble selenium compounds present in nourishment are retained by the gastrointestinal tract (80–95%) [173]. After assimilation, selenium is cleared by the liver and then is carried by selenoprotein P to all organs, with the highest concentrations occurring within the kidney, liver, spleen, testicles, and skeletal muscle [174,175]. Nevertheless, its toxicity has been recorded in livestock for hundreds of years, although the cause is obscure. Within the early 1930s, this ailment, known as “alkali disease”, characterized by the loss of hair and hoof, was distinguished as selenium toxicosis [176,177,178]. Of all the elements, selenium has one of the tightest ranges between dietary insufficiency (<40 µg/day) and toxic levels (400 mg/day) [179], which makes it vital to carefully control intakes by humans and other animals. The suggested dietary remittance is 55 µg/d for persons 14 years or older [180,181]. The sum of selenium accessible in a variable diet with meat, grains, vegetables, and nuts is ordinarily adequate to invalidate the need for supplementation [182]. Seleniferous zones have been pointed in South Dakota (USA), Venezuela, and China [183]. The pathways of selenium toxicity in cellular metabolisms are not yet completely understood. Nevertheless, it is accepted that it can interact with glutathione to form reactive selenotrisulfides and produce oxidative stress, oxidizing cell membranes and macromolecules, compromising cell integrity and thus leading to necrosis or cell death [184]. Different physical forms of selenium lead to different levels of intoxication, for example, the selenide form of selenious acid (H_2_SeO_3_) has an approximately universal fatal outcome [185]. Selenium undergoes an interesting triphasic elimination that depends on its concentration. It is retained in different organ tissues, and its slow clearance system accounts for the permanence of symptoms [180,186,187,188,189].

Selenium is used in several commercial applications which increases the chance of human exposure, namely in solar energy, semi-conductor processing, and in the fabrication of electronics and ceramics [190,191]. It is displayed in steel and copper alloys, used in photographic cells, glass and paint manufacturing, rubber vulcanization, nutritional supplements, and shampoos. Furthermore, it is used alongside different compounds to polish the exterior metallic coating of guns [190,191]. Moreover, the emergence of a debate on the protective effects of selenium against cardiovascular disease [192], myocardial changes [193], and cancer [194] has driven an expansion in the utilization of dietary supplements containing selenium in different organic and inorganic forms [195]. Unfortunately, human mistakes related to fabrication has resulted in harmful concentrations of selenium in these supplements [196,197,198,199]. Occasional cases of acute selenium poisoning are caused by inadvertent ingestion or suicidal attempts [186]. Typical symptoms include intense irritation of the respiratory system and other mucous membranes, metallic taste in the mouth, tingling of the nose and rhinitis. Afterward, edema of the lung and bronchopneumonia can occur. Selenium dioxide (SeO_2_) may produce erythema and necrosis of the skin [200].

**Figure 7:**
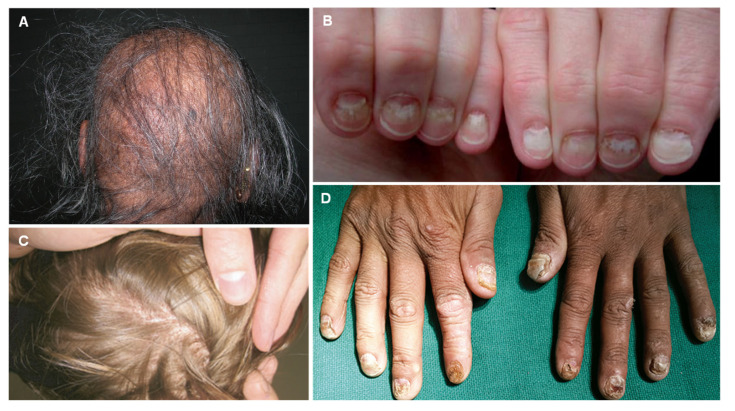
Patchy loss of hair over scalp (**A**). Nail dystrophy 43 days after cessation of the exposure (**B**). Red-brown discoloration of the vertex scalp (**C**). Dystrophy of fingernails (**D**). Reprinted from (**A**)—[210], (**B**)—[211] (**C**)—[212], (**D**)—[209].

Selenosis is the term used for the manifestations of selenium poisoning. Comparing acute and chronic poisoning, selenosis resulting from chronic intoxication often results either from the consumption of organic compounds in seleniferous plants or livestock, supplements, or by occupational exposure to inorganic selenium in an industrial context [191,201]. Most reported cases of acute selenium poisoning are related to industrial accidents via inhalation of selenium dust and fumes or hydrogen selenide, causing irritation of the respiratory tract [191,200,202]. Classic signs and symptoms reported are gastrointestinal complaints, metallic taste and a garlic-like breath odor, alopecia or brittle hair (Figure 7A), nail changes (red pigmentation and paronychia (Figure 7B,D)), lack of mental alertness, dermatitis, mucosal irritation, and “rose eye”—a swelling and pink discoloration of the eyelids. The typical garlic odor of breath and sweat is the consequence of the production of dimethyl selenide and reflects high blood concentrations of selenium [195,200]. More serious presentations include musculoskeletal dysfunction, neuropathy, liver failure, coma, and death [191,200,201,203,204]. The most commonly reported signs in endemic areas of high concentrations of selenium in the soil were alopecia and nail dystrophy [205]. Alopecia has been credited to the disruption of structural proteins in keratin; it is formulated that selenium coordinates into disulfide bridges, causing a structural impairment that leads to hair loss [206]. In contrast to androgen-induced hair loss or alopecia areata, induced alopecia by selenium is generalized [207]. The hair becomes dry and brittle and is easily broken off at the scalp, while the follicles remain intact. Usually, hair can be removed by scratching, this results in a rash on the scalp associated with itching. Hair may also fall from the brows, beard, armpit, and pubic area. New hair is always depigmented and loses its luster. The nails become brittle, and white spots and longitudinal streaks appear on the surface. Thumbs seem to always be affected first. The new nail is also dystrophic, and its surface is rough and stripped. Repeated attacks may lead to acropachy, which is characterized by soft-tissue swelling of the hands and clubbing of the fingers.

Skin lesions have been reported, primarily on the dorsum of hands and feet, lateral side of legs and thighs, the forearms, and the back of the neck. The skin gains a reddish pigmentation (Figure 7C) that usually remains, and can develop to being swollen and then blistered and ulcerated. Dental anomalies such as tooth decay and mottling are also observed [205,208,209].

## 9. Mercury

Mercury (Hg) is a toxic heavy metal [213] and positions around the 67th place in natural abundance among the elements in crustal rocks. It occurs in the environment in three major chemical states: elemental/metallic mercury, inorganic or salts of mercury, and organic or organomercurials (e.g., methyl, phenyl, alkyl) [214], which have diverse pharmacokinetic properties [213,215,216]. Reported use of mercury dates back to 1500 B.C., when the Chinese first mined and used a substance variously known as cinnabar, vermillion, or Chinese red. It is believed that Aristotle called mercury “quick silver,” a title that has held to this day. After that, the Mesopotamians named the planets after metals and Mercury is the only metal that still gives a planet its name [217,218,219]. The metallic form of mercury has also been named as quicksilver or liquid silver, given its liquid, silvery appearance [220]. For more than 3000 years, mercury and its derivatives have been used as cathartics, antiparasitics, antisyphilitics, antipruritics, antiseptics, antiphlogistics, diuretics, vermifuges, dental amalgams, and alternatives [217,218]. In the 1400s, mercury was used in Western Europe as an antisyphilitic, which explains the origin of the phrase ‘‘two minutes with Venus, two years with mercury’’ [221].

Presently, occupational exposure to mercury occurs in more than 60 industries, counting those that manufacture glass thermometers, neon lights, paper, paint, jewelry, insecticides, fungicides, batteries, barometers, chlorine, and caustic soda, as well as those occurring within the realm of dentistry [5]. Environmental exposure to mercury occurs, primarily, from the burning of coal by power plants, resulting in water pollution, and from inappropriate dumping of batteries, paints, lights, and industrial products. As the consumption of fish in the human diet increased, as well as the contamination of water and consequently of these animals, environmental intoxication of Hg has become more relevant [222].

By depleting the thiol reserves in the mitochondria and by binding to intracellular sulfhydryl-containing enzymes and proteins, mercury disrupts normal cell biological roles, resulting in apoptosis [223,224]. Although an overlap in the clinical manifestations of the various forms of mercury is observed, some of these (or combinations of some) are more prevalent in one form of mercury than in the other [220,221,225,226]. Therefore, the most commonly reported clinical syndromes of each form of mercury will be addressed separately.

### 9.1. Elemental Mercury

Elemental mercury is a lipophilic, volatile, heavy, nonwetting liquid that volatilizes to an odorless gas in sufficient amounts to cause clinical toxicity at room temperature [227]. It is known to be one of only two metals that are known to be liquid at room temperature [228]. The vapor pressure of mercury roughly doubles for each 10 °C temperature increment, so that its heating significantly increases exposure and toxicity [3]. The specific characteristics of mercury permitted humans to utilize it in a lot of commercial applications such as thermometers, barometers, thermostats, electronics, batteries, dental amalgams, home folk remedies, and several other uses [225,229].

Toxicity from elemental mercury most commonly emerges from the inhalation of mercury vapors in workplace exposure through the inappropriate dealing of mercury, accidental spills, and poor ventilation. Accidental breakage of mercury-containing devices, such as thermometers, can result in residential exposure [5]. The small amount of Hg(0) in a thermometer can cause intoxication in a warm indoor environment [215]. Elemental mercury combines with sulfhydryl groups on cell membranes and interferes with protein and nucleic acid production, calcium homeostasis, and protein phosphorylation, resulting in oxidative stress and cellular damage [230]. More than 80% of the inhaled elemental mercury vapor is absorbed and diffuses rapidly across cellular membranes, including the blood–brain barrier and placenta [231,232]. Target organs for elemental mercury vapor include the lungs, brain, and to a lesser degree, the kidneys [226,227,229]. In the body, elemental mercury has a long half-life of approximately 60 days [231]. Compared to the respiratory tract, gastrointestinal absorption is insignificant [233,234]. In most cases, cutaneous exposure is also of little clinical consequence [227]. Intravenous mercury infusion is an uncommon source of exposure and occurs in cases of attempted self-harm [235].

Acute toxicity may manifest within hours of a large exposure with gastrointestinal complaints, chills, weakness, cough, and dyspnea, with severe cases resulting in adult respiratory distress syndrome [236] and renal failure [237]. While the lung is the critical organ in acute exposure at very high levels, the central nervous system is the main target organ after chronic exposure to mercury vapors [238]. Chronic mercury toxicity may develop over a period of weeks to months, depending on the level of exposure. Primary symptoms commonly mimic a viral illness [226,239]. Chronic exposure results in two different clinical syndromes: acrodynia and erethism [226,229,239].

**Figure 8:**
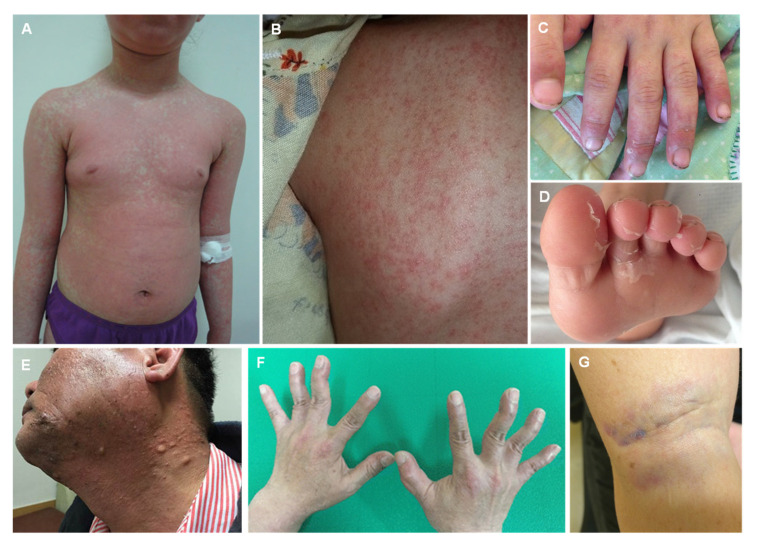
Dermatological manifestations of acrodynia: maculopapular rash in the trunk (**A**,**B**), exfoliation on fingers and pink discoloration of nails (**C**) and toes (**D**), hyperpigmentation with multiple cysts, chloracne-like lesions of face and neck (**E**), swan-neck deformity of both fingers (**F**), bluish gray-stained area on the skin (**G**). Reprinted from (**A**)—[215], (**B**–**D**)—[268], (**E**,**F**)—[269], (**G**)—[270].

“Acrodynia” (in Greek means “painful extremities”), also known as pink disease, Feer syndrome, and Feer-Swift disease, is an uncommon syndrome that typically emerges from elemental mercury exposure, but may also occur from contact with phenyl mercury and mercury salts [240,241,242,243]. This intoxication usually occurs in infants and children since they find the shiny, gray appearance of Hg0 exceptionally appealing [244]. When in contact with elemental mercury through the skin, children can develop acrodynia. Characteristic findings are the following: (i) autonomic changes—sweating, hypertension, tachycardia; (ii) dermatological/dental changes—pruritus, erythematous rash (Figure 8A,B), pink discoloration of nails (Figure 8C), erythematous gingiva, pink hyperpigmentation on the skin (Figure 8E,G), ulceration of oral mucosa, loose teeth; and (iii) musculoskeletal—weakness, poor muscle tone [226,229,245]. The autonomic changes are caused by the capacity of mercury to inactivate the coenzyme S-adenosylmethionine which inhibits the enzyme catechol-o-methyltransferase, creating elevated levels of catecholamines in the organism. The increment in catecholamines causes hypertension, sweating, and tachycardia that may mirror the presentation of a pheochromocytoma [246,247,248]. A 24 h urine sample on these patients uncovers elevated levels of urinary catecholamines, although typically to a lesser degree than that seen in a pheochromocytoma [248].

Patients who have mercury poisoning regularly develop characteristic personality changes collectively named “erethism”. Neuropsychiatric findings include memory loss, drowsiness, lethargy, depression, withdrawal, irritability, insomnia, shyness, confusion, hallucinations, manic-depressive episodes, and emotional lability [229]. Furthermore, incoordination and a fine motor intention tremor primarily involving the hands is also seen in mercury poisonings, accompanied by a rough and rhythmic movement [249,250]. Later, it affects the eyelids, lips, tongue, and head [251,252]. The nervous system suffers the most prominent damage from the chronic accumulation of mercury within the body and generally results from exposure to air mercury concentrations greater than 0.1 mg/m^3^ [253].

The classic triad of intention tremors, erethism and gingivitis is the major manifestation of chronic exposure to elemental mercury vapors. Along with the occurrence of gingivitis, hyperpigmentation is often seen and appears as a blue or black line along the gingival margin [254]. Gingivitis and excessive salivation are the complaints most frequently reported [255,256]. It should be noted that there is a significant clinical overlap between the features of elemental mercury poisoning, especially acrodynia, and those of Kawasaki disease [5].

### 9.2. Inorganic Mercury

Inorganic mercury consists mercuric salts and mercurous salts, the foremost common being mercury sulfide (HgS), commonly known as cinnabar or vermillion [229], and is used as a pigment [226]. Mercurial salts have been utilized in a variety of industries, including medicine as antiseptics (i.e., mercuric chloride), cosmetics, explosives, dyes and pigments, and as antifungals in paints [226,229,257].

Unlike the elemental form, mercury salts are very corrosive to the gut and ingestion is the most common route of poisoning [229,258]. Inorganic mercury is also absorbed promptly through the skin, so patients may develop systemic mercury toxicity even if using only topical preparations [229]. After the ingestion of mercurial salts, patients may complain of oral pain or burning—stomatitis—and other gastrointestinal complaints, such as nausea, vomiting, diarrhea, hematemesis, bloody stools, or abdominal discomfort [229,258]. Colitis with necrosis or sloughing of the gastrointestinal mucosa may develop with severe toxicity, since gastrointestinal tract and the kidneys are the target organs of acute inorganic mercury poisoning. [226,258]. The repetitive cutaneous application can cause hyperpigmentation, swelling, and vesicular or scaly rash. Hyperpigmentation is seen as a gray-brown discoloration being more defined in the skin folds of the face and neck [229]. Similar to elemental mercury, inorganic mercury can result in acrodynia and erethism [226,229].

### 9.3. Organic Mercury

Although the toxicity of elemental and inorganic mercury has been known for centuries, acknowledgment of organic mercury toxicity has been relatively recent due to several major episodes that have brought it to the forefront of environmental toxicology [3]. Methylmercury, an organic mercuric compound, has resulted in the largest number of poisonings and is the most prevalent and toxic form of mercury exposure outside of occupational context [3,5]. In the early twentieth century, organic mercurial compounds were used for industrial and medicinal purposes as preservatives, antiseptics, and seed dressings [1]. Nowadays, the most common source of organic mercury exposure is a dietary consumption of predatory fish through a process known as biomagnification. Inorganic and elemental mercury, from industrial pollution and improper waste disposal, are methylate by soil and marine microorganisms [1,259]. The methylmercury is rapidly absorbed by plankton algae, which are in turn consumed by fish and other aquatic organisms, leading to higher body concentrations of methylmercury concentrations in the tissues of large predatory fish, such as tuna and swordfish [260].

Due to its solubility, approximately 90% of organic mercury is absorbed in the gastrointestinal tract [261]. It promptly crosses the blood–brain barrier and placenta, reaching levels in the brain three to six times higher than those in the blood [262]. Mothers who were exposed to methylmercury while pregnant, gave birth to children that developed congenital Minamata disease characterized by spasticity, seizures, deafness and severe mental deficiency [259,263]. The Food and Drug Administration propose an acceptable daily intake of 0.4 μg/kg body weight/day [263].

Organic mercury poisoning is ordinarily chronic and skin signs are thought to be very uncommon. Nevertheless, mucocutaneous hyperpigmentation can appear in chronic absorption and granulomas can develop from mercury injections directly in the skin. Allergic contact dermatitis is the most common form of mercurial reaction in the skin and can occur by both topical and systemic exposure. In acute poisoning, stomatitis can occur and is believed to represent direct irritation of the mucosa [229]. Neurologic signs and symptoms are common but rarely result in the diagnostic since they may mimic other diseases, including Parkinson’s, Alzheimer’s and depression [264]. The most common presentation of organic mercury poisoning is in the paresthesias of extremities and mouth, incoordination, ataxia, tremor, dysarthria, auditory impairment, and concentric constriction of the bilateral visual fields [259,265]. Autopsy findings commonly report neuronal damage in the gray matter of the cerebral and cerebellar cortex with the most-affected areas being the calcarine region of the occipital lobe and the pre- and postcentral and temporal cortex. In the cerebellum there is a loss of granule cells typically with preservation of the neighboring Purkinje cells [259,266]. Damage of peripheral nerves, largely in the sensory fibers, can occur along with central nervous system impairment [266,267].

## 10. Iron

Iron (Fe) is the most abundant trace element in the body, essential for normal cell metabolism and one of the best-known minerals needed by the human system for homeostasis [271,272]. This element performs a vital role in metabolic processes such as cellular respiration, myelin formation, development of neuronal dendritic trees, and DNA, RNA, and protein synthesis. In addition to this, iron is a co-factor for a myriad of enzymes [273].

The toxicity resulting from intentional or accidental ingestion is a common form of poisoning, especially in the pediatric age group in the context of unintentional ingestion since iron tablets appear as chocolate candy [274,275,276]. Life-threatening toxicity is associated with the pediatric ingestion of potent adult preparations, such as prenatal vitamins [277,278]. The main causes of iron poisoning in adults include suicide attempts and iron overdose during pregnancy [279,280]. Nowadays, iron intoxication is the second most common overdose in pregnancy [281,282], with potentially devastating consequences. In addition, iron toxicity may also occur after multiple blood transfusions for the treatment of chronic disorders such as thalassemia, sickle cell, and hematological cancers [283]. The serum iron, measured between 2 and 6 h after exposure, is the most useful laboratory test, since fast iron distribution from circulation into tissues could result in stabilization of the serum iron level at nearly normal limits [274]. When measured at its peak, levels below 350 micrograms/dL, between 350 to 500 micrograms/dL and above 500 micrograms/dL are associated with minimal, moderate, and severe systemic toxicity, respectively [283].

The absorption of this metal is carefully regulated to avoid accumulation since there is no physiological mechanism to eliminate iron excess from the body. When iron is bound to transferrin, ferritin, or other transport or storage proteins, it is not available to catalyze the formation of free radicals [284]; in patients with an overload of iron, the capacity of these proteins to bind with iron is overwhelmed and transferrin becomes saturated. This results in the increase in free iron ion on the serum, which is directly cytotoxic to almost every organ, leading to tissue damage [275,285]. Iron toxicity is classified as corrosive or cellular. At the cellular level, this element compromises the metabolism in the heart, liver, and central nervous system. Free iron enters cells and concentrates in the mitochondria, disrupting oxidative phosphorylation, catalyzing lipid peroxidation, and forming free radicals, thus leading to anaerobic metabolism and ultimately leading to cell death [274]. When cellular injury occurs, metabolic acidosis is usual [283]. Its corrosive effect can cause direct caustic damage to the gastrointestinal mucosa, leading to a presentation that may mimic an acute abdomen. The recognition of this clinical constellation as a possible presentation of iron poisoning is imperative. Leukocytosis, hyperglycemia, and metabolic acidosis support the diagnosis of moderate to severe iron poisoning but are nonspecific [274,286].

The pathophysiology of iron intoxication was initially described by Covey [287] in 1954. This approach has been modified to include five clinical stages. This exact course may not be found in all patients, and in cases of massive overdose, the patient may present in shock. Determination of the iron toxicity stage should be based on symptoms and clinical manifestations and not on the time of ingestion [274,288]. In stage I, typically occurring between 30 min to 6 h, the patient exhibits gastrointestinal symptoms such as nausea, abdominal pain, vomiting, diarrhea, hematemesis, and hematochezia [274,289,290]. Stage II is characterized by relative stability and occurs between 6 to 24 h. It might correspond to true recovery or to the anticipation of clinical deterioration [274,290,291]. Circulatory shock corresponds to stage III of iron intoxication and emerges typically 6 to 72 h after exposure. Patients may present recurrence of GI symptoms, shock, pulmonary dysfunction, and metabolic acidosis. Iron-induced coagulopathy, hepatic dysfunction, cardiomyopathy, and renal failure are also observed in this stage [281,282,290]. On the IV stage (12 to 96 h), hepatic necrosis may occur and is characterized by an elevation of aminotransferase levels and possible progression to hepatic failure [281,282,290]. Hepatotoxicity is associated with 50% of mortality [292] and it is found to be dose-dependent [293]. In the last stage, stage V, the consequences after an acute injury to the gastrointestinal tract are highlighted. The healing process may include pyloric or proximal bowel scarring and obstruction, with areas of stenosis. These late consequences rarely occur [280,281,282,290,294].

**Figure 9:**
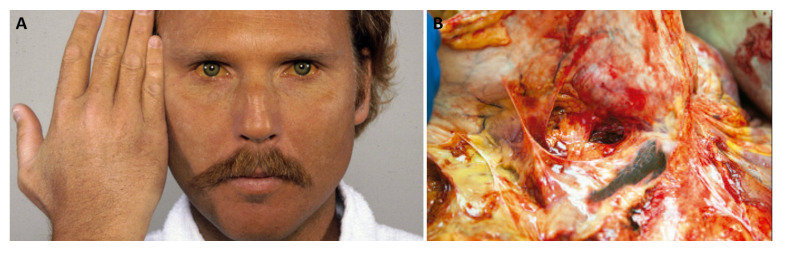
Skin hyperpigmentation—bronze coloration (**A**), a black-greyish, rigid pancreas (**B**). Reprinted from (**A**)—[313] and (**B**)—[314].

Hereditary hemochromatosis involves a number of chronic iron overload syndromes characterized by excessive parenchymal iron deposition (Figure 9B), leading to tissue injury and dysfunction in several organs [295,296]. It was first described in 1865 by Trousseau [297], when he followed a patient with liver cirrhosis, diabetes mellitus, and skin hyperpigmentation—the so called “bronze diabetes”. Hereditary hemochromatosis is recognized as the most frequent recessive autosomal disease in European origin population, namely of Celtic origin [296]. Approximately 10% of Caucasians in Western countries carry one gene for hemochromatosis [298]. It is classified into two main groups: HFE mutations and non-HFE mutations [296]. When the HFE mutation is present, this syndrome is known by hemochromatosis type I or classic hereditary hemochromatosis and represents 90% to 95% of all cases [299]. The HFE gene is located on chromosome 6 near the HLA locus with a homozygous C282Y mutation most of the time [295]. The HFE gene controls intestinal iron absorption and deposition in the tissues, especially the liver, heart, pancreas, pituitary gland, joints, and skin [300]. Non-HFE mutations include hereditary hemochromatosis type II, III, and IV. Hereditary hemochromatosis type II is the juvenile form, which is related to an alteration of the hepcidin protein that regulates the absorption of iron [301]. Juvenile form appears in the first three decades of life and has a more severe phenotype than classic hereditary hemochromatosis [302]. Hereditary hemochromatosis type III is caused by mutation of the type 2 transferrin receptor gene and type IV to a mutation in the slc40 gene, which encodes the ferroportin gene: a major transporter of cellular iron [303,304]. Type IV, unlike the other types, has a dominant form of transmission [304].

The symptoms and signs are dependent on the insidious accumulation of iron, which occurs steadily and slowly over decades. Most of the patients are asymptomatic until later in life; men develop signs and symptoms between the third and fifth decade, and women after the age of 50 due to lactation and the physiological blood losses that occur during menstruation and pregnancy [305,306,307,308,309,310,311]. The most common and early symptoms include fatigue (70% to 80%), arthralgia/arthritis (40% to 50%), abdominal pain (20% to 60%), decreased libido or impotence (20% to 50%), and weight loss (10% to 50%). The most frequent clinical symptoms for diagnosis are hepatomegaly (50% to 90%), skin hyperpigmentation (Figure 9A)—a bronze or slate-grey coloration (30% to 80%), hypogonadism (20% to 50%), arthropathy, splenomegaly, diabetes mellitus, cirrhosis, cardiomyopathy and arrhythmia [305,306,307,308,309,310,311,312]. In advanced stages of the disorder, the dysfunction and tissue injury leads to diabetes mellitus, cirrhosis, congestive heart failure, cardiac arrhythmia, and liver cancer [312].

## 11. Cadmium

The heavy metal cadmium (Cd) is known to be an ubiquitous trace element and environmental contaminant [315,316]. This element is one of many metals that are not physiologically or biochemically essential to organisms [317]. Cadmium occurs naturally in ores together with zinc, lead, and copper. Although the incidence of chronic cadmium poisoning in industrial workers declined in the second half of the last century, its compounds are still widely used in industries in spite of an increased awareness of the effects of chronic exposure to cadmium [318]. Currently, cadmium is used in the production of anticorrosive agents: as a stabilizer in polyvinyl chloride (PVC) products, as a color pigment and neutron absorber in nuclear power plants, and, now most commonly, in the fabrication of nickel-cadmium batteries. Cadmium is also present as a pollutant in phosphate fertilizers [318,319,320]. Compared with mercury or lead, cadmium passes more easily from soil to plants and can readily enter the food chain. In addition to this, the uptake process by plants is enhanced at low pH [321].

Its biohazardous potential was first described in 1817 by Friedrich Stromeyer in those working in zinc smelters. In 1948, emphysema and proteinuria in industrial workers exposed to Cd dust was described by Friberg, linking cadmium exposure to renal toxicity [319]. Pulmonary, gastrointestinal, and dermal absorption of cadmium is possible, cigarette smoking being a major source of cadmium exposure [322]. Indeed, the concentration of cadmium in smokers is 4–5 times higher in blood, and 2–3 times higher in the kidneys, when compared with non-smokers [321]. In non-smokers, food is the most important source of cadmium exposure, from contaminated soil and water [323]. In the liver, cadmium induces the production of metallothionein [324]. The cadmium-metallothionein complex (Cd-MT), is freely filtered at the glomerulus and reabsorbed by the proximal tubule leading to a very long half-life (approximately 10 to 30 years). This complex formation is a protective response to limit toxicity from free cadmium, but when its production is exhausted by chronic exposure to cadmium, the intracellular levels of this element increases, accumulates in mitochondria, and inhibits the respiratory chain by acting at the level of complex III [325]. During every person’s lifetime, the quantity of Cd in the kidney tubular cells increases [326]. The tissues most affected by chronic cadmium toxicity are the kidney, skeleton, and lungs. Approximately 50% of the accumulated dose is stored in the kidneys, which makes the kidney the main organ for long-term cadmium accumulation [327,328]. The primary marker of kidney damage is evidenced by an increased excretion of low molecular weight proteins such as β_2_-microglobulin (β_2_M) and retinol-binding-protein (RBP), and enzymes such as N-acetyl-α-D-glucosaminidase (NAG) [321,323,329,330]. This is called “tubular proteinuria” and represents a good index of proximal tubular damage [320]. The urinary concentration of cadmium reflects past exposure, body burden, and renal accumulation, while blood concentration serves as a reliable indicator for a recent exposition [331].

The most important metabolic parameter for cadmium uptake is a person’s possible deficiency of iron. People with low iron reserves showed a 6% higher uptake of cadmium than those with a normal iron supply [332]. This may explain why children or menstruating women tend to have higher cadmium levels [333] when compared with men who have experienced similar levels of environmental exposure. Low iron blood levels stimulate the expression of the divalent metal ion transporter 1 (DCT-1) of the gastrointestinal tract, increasing cadmium absorption [334].

Acute intoxication is now rare in Europe and North America and its symptoms depend on the route of ingestion. Acute exposure to cadmium fumes leads to a shortness of breath, lung edema, and destruction of mucous membranes as part of cadmium-induced pneumonitis [335,336]. The intake of cadmium-contaminated food causes desquamation of the intestinal mucosa, resulting in severe and bloody diarrhea and vomiting [337,338]. The association between cadmium and skeletal damage was first described in 1950, in Japan, where the exposure was caused by cadmium-contaminated water used for the irrigation of local fields [321,339]. This disease became known as Itai-Itai [340] and the affected population showed a wide range of symptoms such as a low grade of bone mineralization, a high rate of fractures, an increased rate of osteoporosis, and intense bone pain (a combination of osteomalacia and osteoporosis) [341]. Exposure to cadmium-polluted fume and dust (e.g., in industry workers) may also lead to the development of bone damage [342]. During recent years, new data suggest that relatively low cadmium exposure is enough to give rise to skeletal damage, as evidenced by osteoporosis and fractures [343,344,345]. In addition to the effects on the kidney and bones, chronic cadmium toxicity causes infertility [346] and, more recently, has been linked to a potential cardiovascular risk factor, shown by an increased risk of myocardial infarction [347], development of hypertension [348], and diabetes mellitus type 2 [349].

## 12. Bismuth

Bismuth (Bi) is a trivalent poor metal that chemically resembles arsenic and antimony [350,351]. It is thought that its designation derives from the German word “wismuth” (white mass). It is the least abundant element of the fifteenth group and a relatively rare element. Bismuth is naturally monoisotopic (209Bi) and is considered to be the heaviest stable element since its theoretical half-life is 1.9 × 10^9^ years. It is sourced as a by-product of lead, copper, and tin mining, being primarily found as bismuthinite (bismuth sulfide) and bismite (bismuth oxide) ores [352]. Until today, no natural biological role of this metal was found. There are references to the uses of inorganic and organic bismuth compounds in medicine dating back to the late 18th century [353]. They have been used in a variety of diseases such as skin conditions and the treatment of war wounds, through topical application (as astringents, emollients, and antimicrobials), syphilis by parenteral administration, and orally to cholera infantum, peptic ulcer, and other gastrointestinal disorders [354,355,356,357]. Although the demand for these components declined dramatically after the appearance of antibiotics, for example in the treatment of syphilis in the 1980s, with the discovery of *Helicobacter pylori*, bismuth emerged again as an effective treatment for many gastrointestinal complaints [350,351,358,359]. Popularized in the First World War, bismuth iodoform paraffin paste (BIPP) gauze is an antiseptic dressing [360] still used frequently by maxillofacial surgeons and otolaryngologists. Furthermore, bismuth iodoform paraffin paste is used in the management of epistaxis [361]. However, neuropsychiatric symptoms can develop from the use of bismuth iodoform paraffin paste gauze. In order to prevent it, clinicians should take into account the patient’s renal and hepatic function as well as the size and condition of the wound [362].

Absorption is primarily via the gastrointestinal tract [363], and although organic bismuth compounds show minimal absorption, it is still significant. Inorganic compounds with poor solubility are not absorbed [364,365,366]. Solubility is increased in the presence of hydroxy and sulfhydryl groups and by the acidity of the medium. Moreover, it seems as though the absorption of bismuth is of high inter- and intraindividual variation [367,368,369].

After bismuth enters the bloodstream, it is distributed into all tissues with high concentrations and toxicity in the kidneys, but also in the liver [363]. In the kidney, bismuth binds to a metal-binding protein in proximal renal tubule cells and remains bound for months. Its toxicity is attributed to its affinity to combine with sulfhydryl groups of vital enzymes, leading to destroying the functions of these enzymes [364,367]. Since greater than 99% of ingested bismuth is not absorbed, it makes it suitable as a locally acting gastrointestinal agent [370]. The residual compounds that are absorbed are eliminated unmetabolized by the urinary and hepatobiliary routes. The exact proportion contributed by each route is still a subject of discussion [367,371]. Taking therapeutic bismuth compounds can lead to serum levels between 10–20 µg/L, and serum levels greater than 50 µg/L can be a sign of toxicity (normal < 0.5 μg/L) [364,367]. The reported toxic effects attributed to bismuth poisoning include hepatitis and hepatic fatty degeneration [372], nephropathy [373], osteoarthritis [367], gingivostomatitis [370], colitis [364] and several neuropsychiatric symptoms [363,370,374,375].

Generally, acute bismuth toxicity (single high dose) manifests as nephrotoxicity, whereas chronic exposure to high levels of bismuth salts result in encephalopathy [350,364]. Severe overdose of acute bismuth induces reversible proximal tubular damage, leading to acute tubular necrosis. Acute tubular necrosis results in proximal tubular dysfunction (Fanconi’s syndrome), which is clinically presented by hypophosphatemia, hypouricemia, metabolic acidosis, renal glycosuria, and tubular proteinuria. Bismuth is known to cause a reversible Fanconi’s syndrome and to follow the path of acute tubular necrosis [376,377]. Several heavy-metal intoxications are described to cause reversible Fanconi’s syndrome, such as bismuth, lead, and mercury [378], unlike chronic cadmium intoxication, which has been linked with irreversible Fanconi’s syndrome [379]. Bismuth crosses the blood–brain barrier and binds to enzymes involved in oxidative metabolism, resulting in a reduced oxygen consumption and cerebral perfusion. This can lead to neuropsychiatric symptoms and encephalitis with brain lesions [364,380]. There are several reports of neurotoxicity from the use of bismuth iodoform paraffin paste impregnated gauze since a well-study outbreak of bismuth encephalopathy in 1970 established the typical symptoms of oral bismuth toxicity to be depression, anxiety, irritability, and possibly mild incoordination [369]. Through the years, several clinicians reported that more neuropsychiatric symptoms would develop after procedures with the use of BIPP gauze: malaise, insomnia, personality change [360], ataxia [381], dysphonia [382], dysarthria [383], gait dyspraxia [382], myoclonic jerks [384]—more common in the distal upper limbs [367], delirium, drowsiness [384], and coma [369].

**Figure 10:**
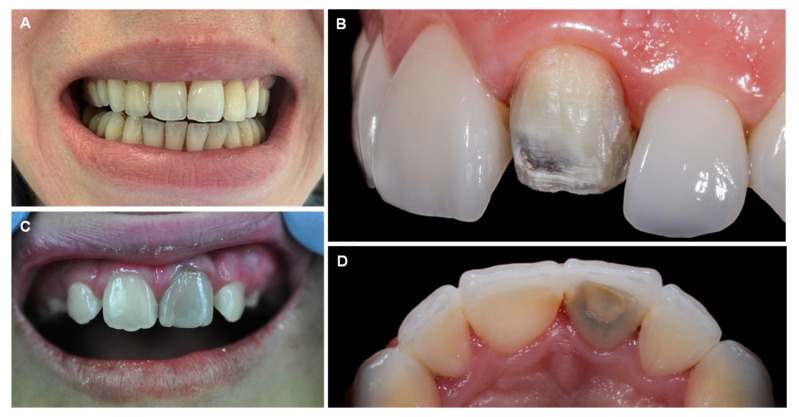
Greyish discoloration of teeth—(**A**–**D**). Reprinted from (**A**)—[392], (**B**,**D**)—[393] and (**C**)—[394].

Another characteristic feature of bismuth ingestion is the gingivostomatitis with bluish black gum discoloration, gingivitis, and ulceration [385,386,387,388,389]. Chronic intoxication from repeated oral or parenteral doses causes “ bismuth line”, a gum condition with black spots of buccal and colonic mucosa [390]. Blackening of the tongue and teeth (Figure 10A–D) has been reported as a harmless side effect of bismuth toxicity [391]. It has been suggested that the oral lesions may be secondary to the nephrotoxic effect of the bismuth compounds [387,388].

## 13. Conclusions and Future Perspectives

Heavy metals are dense, naturally occurring elements that accumulate in the environment, mostly due to anthropogenic industrial activities, causing health hazards for humans and other creatures [395,396]. Environmental pollution can result in the contamination of air, water, sewage, seawater, waterways, and can accumulate in plants, crops, seafood, and meat and indirectly affect humans [395]. Some occupations, such as metal finishing industry workers and traditional glassworks, have increased the risk for exposure and toxicity of a particular chemical element [397]. The clinical course of intoxication is determined by the chemical element involved, the level and mode of exposure, the chemical and valance states of the compound (i.e., elemental, organic or inorganic), and due to interindividual variability of the patient [1,397]. Major signs and symptoms associated with poisoning by the addressed chemical elements in this review are compiled in Table 1.

Despite the several sources and routes (e.g., inhalation, ingestion, or skin contact) of exposure, intoxication by chemical elements is still underestimated in the global medical community. Mining, fossil-burning, agriculture, seafood, consuming plants and meat that accumulate these metals from contaminated water and soil are the most common natural causes of toxicity. The incidence and magnitude of the toxicities are dependent on the natural soil content, geographical location, location and number of industries, regulatory measures to contain pollution, healthcare facilities, and individual factors such as genetics and nutritional status [398]. Although accidental exposure is far more common, intentional poisoning has been reported in suicide and homicide attempts [1,9,10,11,12,13]. Bernardino Ramazzini (1633–1714), known as the father of occupational medicine, formulated an injunction that remains the key to healthy occupational practice: “what is your occupation?” [4,84].

In general, the diagnosis of exposure includes the clinical history, physical findings and analysis of samples such as blood, urine, skin, nails, hair, and gastric contents. Of these, the urine sample is typically the most useful for qualitative detection [3,398,399]. Acute toxicity which results from exposure to large doses, within a short time exposure, as well as its presentation are usually dramatic. The most common presentations vary with the form of exposure: respiratory symptoms in cases of inhalation, skin lesions in topical contamination, and, in cases of ingestion, typically mimic acute gastroenteritis. In contrast, chronic poisoning is usually more difficult to detect, requiring a higher level of suspicion. Since chronic poisoning relies on cumulative exposure, scrupulous analyses of the patient history and routine are essential, considering that the recognition of the metal in question is facilitated if the source is known. When unrecognized, acute and chronic toxicity from exposure to heavy metals pose significant morbidity and mortality since treatment is delayed [1]. Management includes the removal of the source and the offending agent (sometimes using chelating agents), supportive therapy, and prevention of further exposure [1,3,398,400].

Hereupon, preventive measures, principally in industrial workers and others at higher risk, such as monitoring air, water, food, screenings of blood and urine for toxicity from heavy metals, should be realized periodically by health authorities [401]. Furthermore, in order to prevent or minimize exposure to populations, cooperations between health and civic authorities must exist and industries must be sensitized for the existence of a social responsibility with regard to proper wastes disposal.

## Figures and Tables

**Table 1:** Signs and symptoms associated with poisoning by the addressed chemical elements.

Mercury (Hg)
Acrodynia: pruritus, erythematous rash, pink discoloration of nails, erythematous gingiva, pink hyperpigmentation on the skin, ulceration of oral mucosa. Erethism: memory loss, lethargy, depression, irritability, insomnia, confusion, hallucinations. Gingivitis Tremors and paresthesia
Thallium (Tl)
Alopecia Palmar and solar keratosis Glossitis and stomatitis Mee’s lines in the nails Bayonet hair
Fluorine (F)
Dental fluorosis Skeletal fluorosis: diffuse bone condensation and interosseous membrane calcification, osteosclerosis and osteoporosis.
Arsenic (As)
Palmar and solar keratosis Hyperpigmentation Mee’s lines in the nails Raindrop pigmentation
Iron (Fe)
Hemochromatosis: skin hyperpigmentation—a bronze or slate-gray coloration.
Selenium (Se)
Alopecia Nail dystrophy/Paronychia Pink pigmentation of nails and eyelids Dermatitis and skin lesions Reddish pigmentation of the skin
Bismuth (Bi)
“Bismuth line”: bluish black gum discoloration Gingivostomatitis and ulceration Blackening of the tongue and teeth
Cooper (Cu)
Kayser–Fleischer ring Sunflower cataract
Lead (Pb)
Burton’s line in the gums Lead lines on X-ray of long bones
Cadmium (Cd)
Osteomalacia and osteoporosis Long exposure causes anosmia

## Data Availability

Not applicable.
